# Pathology of Angina Pectoris

**Published:** 1871-03

**Authors:** 


Pathology of Angina Pectoris.—MM. A. Eulenburg and P. Guttman discuss (*Archiv fur Psychiatrie*, t. ii 1869) all the facts known and the theories advanced in regard to this affection, and they conclude that it is a neurosis both of motion and sensation. The symptoms to which it gives rise may be excited by causes of a different nature, even extraneous to the heart. All the cardiac nerves are probably more or less affected in this disease, and the variation in the phenomena observed in different individuals results doubtless from the more or less active part taken by the nerves which unite together in the cardiac plexus in the production of these phenomena. It is probable that the *great sympathetic* performs the most important part, inasmuch as it constitutes the larger portion of the cardiac plexus.—*Archives Generales*, Sept., 1870.

